# Serum *NEAT1/MEG3* and *miR-124/miR-146a* Dysregulation in Behçet’s Disease: Association with Th17/NF-κB-Related Inflammation and Diagnostic Potential

**DOI:** 10.3390/ijms27156720

**Published:** 2026-07-27

**Authors:** Abdullah F. Radwan, Hanan A. Rizk, Mona Sami Awed, Manar R. Senosi, Ahmed Gamal, Olfat G. Shaker, Ghada Ayeldeen

**Affiliations:** 1 Department of Biochemistry, Faculty of Pharmacy, Egyptian Russian University, Badr City 11829, Egypt; abdullah-fathy@eru.edu.eg (A.F.R.);; 2 College of Humanities and Sciences, Ajman University, Ajman 346, United Arab Emirates; 3 Department of Rheumatology and Rehabilitation, Faculty of Medicine, Fayoum University, Fayoum 63514, Egypt; 4 Department of Andrology, Sexology and STI’s, Faculty of Medicine, Cairo University, Cairo 11562, Egypt; 5 Department of Medical Biochemistry and Molecular Biology, Faculty of Medicine, Cairo University, Cairo 11562, Egypt

**Keywords:** Behcet’s disease, long non-coding RNA, microRNA, *NEAT1*, *MEG3*, *miR-124*, *miR-146a*, IL-17, NF-κB, biomarkers, diagnostic performance

## Abstract

Behçet’s disease (BD) is a chronic, multisystem vasculitis characterized by dysregulated immune responses and prominent Th1/Th17 polarization; however, the upstream epigenetic regulatory mechanisms driving this inflammatory imbalance remain incompletely understood. Here, we investigated the coordinated expression and diagnostic relevance of key long non-coding RNAs (lncRNAs; *NEAT1* and *MEG3*) and microRNAs (*miR-124* and *miR-146a*), and their association with IL-17/IL-6-mediated inflammation in BD. In a case–control study, serum levels of the selected non-coding RNAs were quantified by quantitative real-time PCR, while cytokine concentrations were measured using ELISA, complemented by bioinformatic interaction analysis and integrated statistical and machine learning approaches. Patients with BD exhibited marked downregulation of *NEAT1*, *MEG3*, *miR-124*, and *miR-146a* (*p* < 0.0001), accompanied by significantly elevated IL-17 and IL-6 levels. Individually, biomarkers demonstrated strong discriminatory capacity (AUC 0.83–0.92), while combined panels further improved classification performance. Multivariate modeling identified these non-coding RNAs as independent predictors of BD, and machine learning analysis identified *miR-146a* and IL-17 as the most influential contributors to disease classification. Notably, selected biomarkers showed associations with specific clinical manifestations, supporting their potential clinical relevance. Bioinformatic analyses identified putative interactions between *NEAT1* and *miR-124*/*miR-146a*, while *MEG3* demonstrated independent diagnostic value without evidence of direct interaction with the investigated miRNAs. Collectively, these findings provide a hypothesis-generating framework for future mechanistic investigations and support the potential diagnostic value of the investigated biomarker panel, although validation in larger multicenter cohorts and disease-control populations is warranted.

## 1. Introduction

Behçet’s disease (BD) is a chronic, relapsing, multisystemic inflammatory vasculitis characterized by recurrent oral and genital ulcers, skin lesions, ocular involvement, and potentially life-threatening vascular and neurological manifestations [1]. The pathogenesis of BD remains incompletely understood, involving a complex interplay of genetic, environmental, and immunological factors [2]. It is currently classified as “MHC-I-opathy”, exhibiting characteristics of both autoinflammatory and autoimmune disorders [3].

The immunopathogenesis of BD is characterized by dysregulated innate and adaptive immune responses [4]. Key immunological abnormalities include hyperactivation of neutrophils, enhanced T-cell and monocyte responses, altered cytokine production, and macrophage polarization toward a pro-inflammatory M1 phenotype [5]. The disease manifests through a skewed cytokine profile initiated by Th1 polarization and the robust production of interferon-gamma (IFN-γ) and IL-12, allowing for macrophage activation and cytotoxicity [6,7]. However, the defining feature of BD is the proliferation of T helper 17 lymphocyte (Th17) cells [8]. Inflammation is induced by pro-inflammatory cytokines like IL-6 in this subset of T-cells, causing the profound influx of neutrophils in mucocutaneous and ocular manifestations [9]. Pro-inflammatory cytokine dysregulation is aggravated by the important deficiency of regulatory T-cells [10].

In terms of immune system regulation, non-coding RNAs (ncRNAs) have been identified as key regulators of cellular homeostasis. Long non-coding RNAs (lncRNAs) have been identified as a scaffold or interface in the regulation of gene expression [11,12]. The *Maternally Expressed Gene* 3 (*MEG3*) and *Nuclear Paraspeckle Assembly Transcript 1* (*NEAT1*) lncRNAs have been identified in the context of immune cell differentiation and endothelial cell integrity [13,14]. However, the interaction between lncRNAs and the microRNA (miRNA) network in BD vasculitis remains unclear.

The molecular factors that maintain the Th17/Treg imbalance are more complex than just cytokine signaling. New evidence suggests that genetic control is significant in maintaining immune homeostasis at the post-transcriptional level [15]. MicroRNAs (miRNAs) are small, non-coding RNA molecules of 18–24 nucleotides that play fundamental regulatory roles in gene expression [16]. Several studies have identified specific miRNA signatures that distinguish BD patients from healthy controls and correlate with disease activity [17]. These dysregulated miRNAs regulate pathways central to BD pathogenesis, including tumor necrosis factor (TNF), interferon-gamma (IFN-γ), and vascular endothelial growth factor (VEGF) signaling cascades [18].

*MiR-124* and miR-146a are recognized as potent anti-inflammatory regulators [19,20]; *MiR-124* is a known repressor of signal Transducer and Activator of Transcription 3 (STAT3), the main transcription factor for Th17 differentiation, while miR-146a acts as a negative feedback brake on the nuclear factor kappa-light-chain-enhancer of activated B cells (NF-κB) pathway by targeting Tumor Necrosis Factor Associated Factor 6 (TRAF6) [21,22]. The concurrent dysregulation of these miRNAs may contribute to disruption of key immunoregulatory pathways, potentially facilitating the exaggerated inflammatory response characteristic of active BD.

The current study aims to determine the molecular signature of Behçet’s disease by evaluating the co-expression of lncRNAs (*MEG3*, *NEAT1*) with miRNAs (*miR-124*, *miR-146a*) in the context of pro-inflammatory cytokines IL-17/IL-6.

## 2. Results

### 2.1. Demographic and Clinical Diagnostic Data

The results of the present study demonstrate that the control group has been matched for age and gender to the patients with BD. As shown in Table 1, the study population comprises 60 patients with BD and 60 healthy controls. There were no statistically significant differences in terms of age (33.8 ± 7.9 years compared to 35.17 ± 6.06 years; *p* = 0.29) or gender distribution (*p* = 0.157). The clinical characteristics and activity of the 60 BD cases are shown in Table 2. Arthralgia (55%) and ocular (58.3%) were the most common active disease manifestations. The constellation of mucocutaneous manifestations was most commonly oral ulcers, which occurred in 41.7% of all patients with any mucocutaneous symptoms, followed by erythema nodosum (25%), and both genital ulceration and skin pustules were each observed in 13.3% (*n* = 8) of the cohort. Headache was noted in 28.3%. Less common systemic features included gastrointestinal symptoms such as nausea, vomiting, and abdominal pain, noted in 21.7% (13 out of 60), and central vessel involvement, noted in 11.7% (seven out of 60). The least common features included new active nervous system involvement and diarrhea/blood per rectum, each noted in 8.3% (five out of 60), as well as frank arthritis, noted in 6.7%. Disease activity was also quantitatively assessed, with a mean patient’s BDCAF index score of 2.92 ± 0.24. The BSAS was 27.48 ± 2.36, and the mean transformed index of BDCAF was 6.1 ± 0.36. The patient’s global disease activity was higher than the physicians’ assessment (6.48 ± 0.43 versus 5.68 ± 0.42).

### 2.2. Relative Expression of the Studied RNAs in Serum

**Figure 1:**
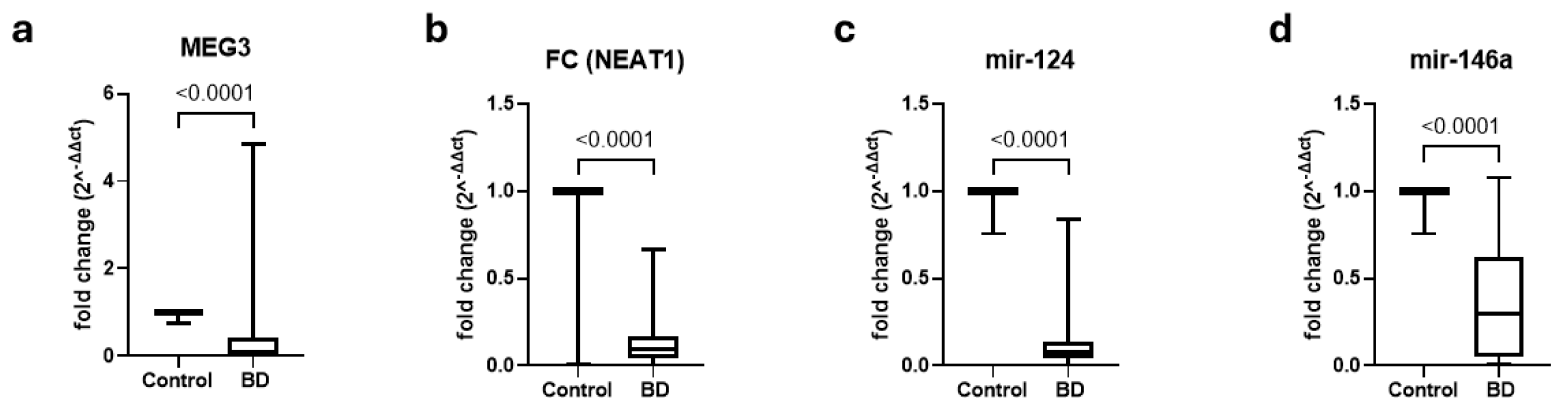
Relative expression levels of non-coding RNAs in the studied groups. Box plots for the fold change expression (2^−ΔΔCt^) of (**a**) lncRNA *MEG3*, (**b**) lncRNA *NEAT1*, (**c**) *miR-124*, and (**d**) *miR-146a* in sera obtained from BD patients (*n* = 60) and healthy volunteers (*n* = 60). The horizontal lines inside the box plots represent the median expression, while the lines extending from the box represent the range. *p* < 0.0001: Significant difference as compared to the control group. BD: Behçet’s disease; Ct: cycle threshold; *MEG3*: *Maternally Expressed Gene 3*; miR: microRNA; *NEAT1*: *Nuclear Paraspeckle Assembly Transcript 1*.

As illustrated in Figure 1 and Table 3, the relative levels of expression of *MEG3* and *NEAT1* were significantly downregulated in patients with BD compared with controls (0.41 ± 0.11 vs. 0.99 ± 0.004 and 0.12 ± 0.01 vs. 0.97 ± 0.017, respectively; *p* < 0.0001). In addition, the expression levels of the microRNAs *miR-124* and *miR-146a* were significantly downregulated in patients with BD compared with controls, with *p*-values of <0.0001 for both.

### 2.3. Serum Levels of IL-6 and IL-17

**Figure 2:**
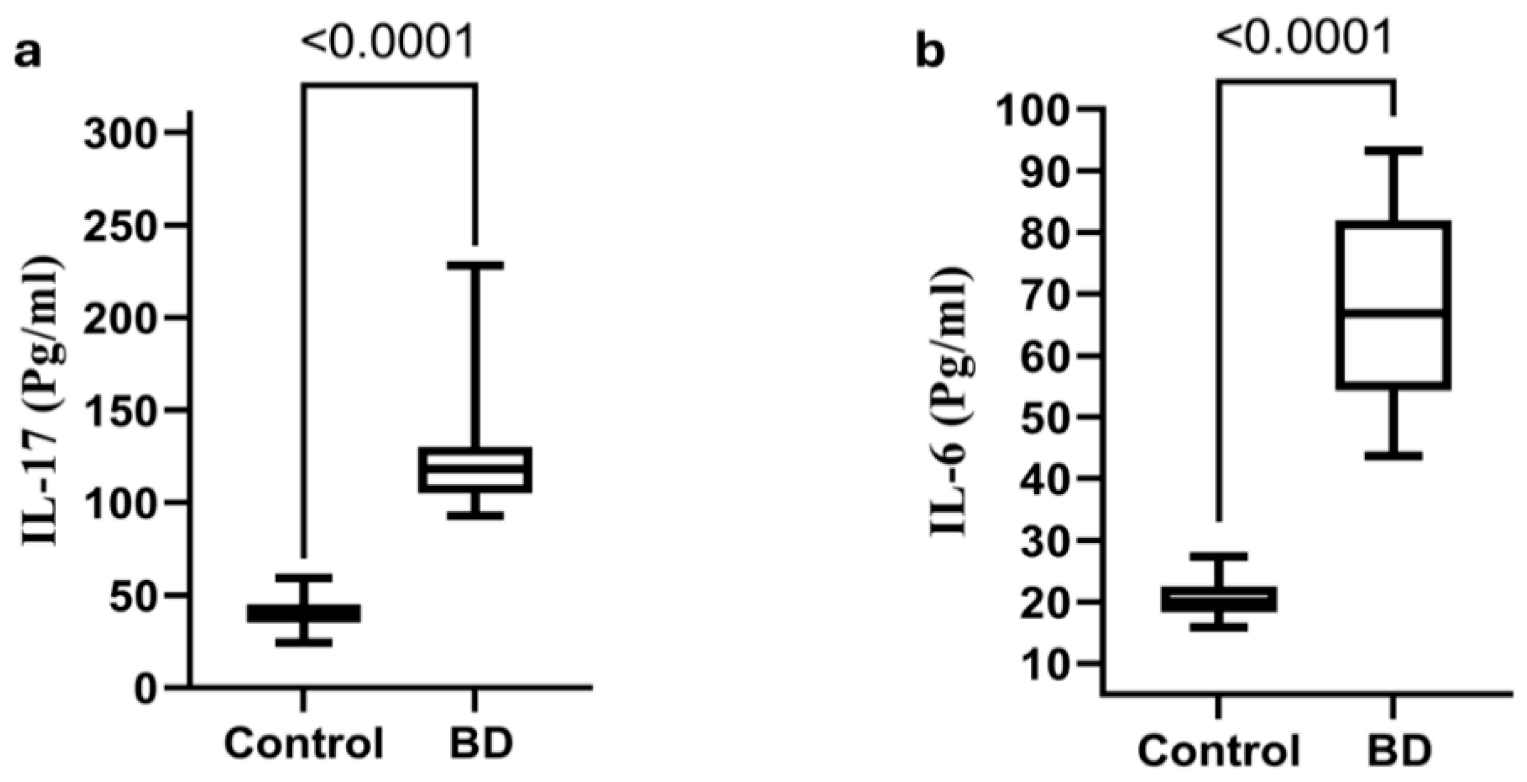
Comparison of serum pro-inflammatory cytokine levels between study groups. Box plots for the serum levels (pg/mL) of (**a**) Interleukin-17 (IL-17) and (**b**) Interleukin-6 (IL-6). The horizontal line within each box plot represents the median, and whiskers represent the minimum and maximum values for each cytokine. *p* < 0.0001: Significant difference versus control group.

As shown in Figure 2 and Table 3, the serum concentrations of IL-17 and IL-6 were significantly higher in the patient group than in healthy controls. The quantitative analysis demonstrated that the level of IL-17 was significantly higher, almost 3-fold, in the patient group than in healthy controls. The level was 134.7 ± 7.92 pg/mL, while it was only 39.62 ± 0.94 pg/mL in healthy controls. Similarly, the level of IL-6 was significantly higher, almost 3-fold, in the patient group than that in healthy controls. The level was 67.81 ± 2.01 pg/mL, while it was only 20.62 ± 0.46 pg/mL in healthy controls.

### 2.4. Association Between Serum Biomarkers and Clinical Manifestations

To determine whether specific clinical phenotypes were associated with certain biomarker profiles, patients were divided into groups with and without clinical manifestations and were analyzed using the Mann–Whitney U test (Table 4). In general, the levels of the studied non-coding RNAs and cytokines were not significantly different between genders and between the presence and absence of mucocutaneous (oral and/or genital ulcers, erythema nodosum, and skin pustules) and systemic (articular, neurological, vascular, and gastrointestinal) manifestations (*p* > 0.05). However, there was a significant association with ocular manifestations. Patients with new active eye manifestations were shown to have significantly lower levels of *MEG3* (0.07; IQR: 0.03–0.21) than those without ocular manifestations (0.25; IQR: 0.06–0.60) (*p* = 0.01). There was no significance with the other biomarkers and ocular manifestations.

### 2.5. Correlation Analysis Between Serum Biomarkers and Disease Activity Scores in BD Patients

To assess the possibility of utilizing serum biomarkers as severity indicators of the disease, Spearman’s rho correlation analysis was conducted for the relationship of the levels of the investigated biomarkers with clinical disease activity indices (Table 5). Among the investigated parameters, it was observed that serum levels of IL-17 were positively correlated with the BDCAF Patient’s Index Score (r = 0.274, *p* = 0.03). However, no significant correlation was observed for serum levels of IL-17 with the transformed BDCAF score or BSAS. In contrast, serum levels of IL-6, as well as the expression levels of the non-coding RNAs *MEG3*, *NEAT1*, *miR-124*, and *miR-146a*, were not associated with the disease activity scores, BDCAF, or BSAS, as all *p* values were greater than 0.05.

### 2.6. The Study of Correlation Between Serum Biomarkers, Clinical Phenotypes and Disease Activity Indices

**Figure 3:**
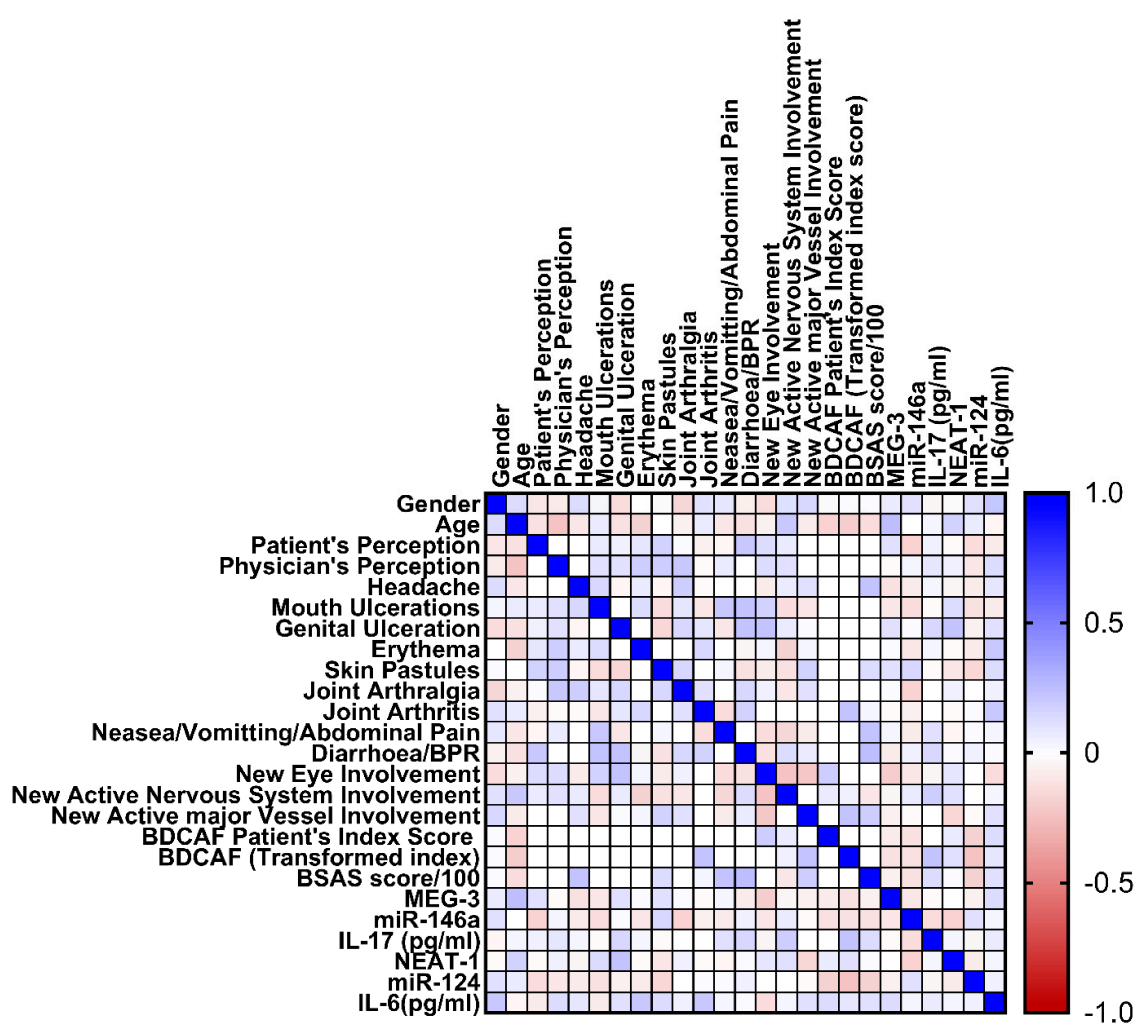
The relationship between serum biomarkers and clinical manifestations and disease activity indices in BD patients. Heat map shows Spearman’s rho correlation coefficients of the variables under study. The relationship between the variables is conceived as follows: Blue: positive correlation; red: negative correlation; white: no correlation. The intensity of the different colors is correlated to the magnitude of the corresponding correlation coefficient, which varies from −1.0 to +1.0. Abbreviations: BDCAF: Behçet’s Disease Current Activity Form; BPR: bleeding per rectum; BSAS: Behcet’s Syndrome Activity Score; IL: Interleukin; *MEG3*: *Maternally Expressed Gene 3*; miR: microRNA; *NEAT1*: *Nuclear Paraspeckle Assembly Transcript 1*.

Spearman’s rho correlation analysis revealed different patterns of correlations between the serum biomarkers, clinical features, and demographic factors (Figure 3). It was found that the presence of serum IL-17 was positively associated with systemic inflammatory activity. It was positively correlated with Joint Arthritis (r = 0.358, *p* <0.01), New Active Major Vessel Involvement (r = 0.276, *p* <0.05), Joint Arthralgia (r = 0.265, *p* <0.05), and BDCAF Patient Index Score (r = 0.274, *p* <0.05), while being negatively correlated with *MEG3* (r = −0.42, *p* < 0.01). Interestingly, the expression profile of *miR-124* was also tissue-specific, as reflected by the moderate positive correlation with New Active Nervous System Involvement (r = 0.337, *p* <0.01) and negative correlations with New Eye Involvement (r = −0.275, *p* <0.05) and Joint Arthralgia (r = −0.260, *p* <0.05). At the demographic level, younger Age was associated with higher levels of disease activity in specific domains, as reflected by negative correlations between Age and the expression levels of miR-146a (r = −0.287, *p* < 0.05) and Skin Pustules (r = −0.296, *p* < 0.05). At the molecular level, a moderate positive correlation was observed between the long non-coding RNAs, *MEG3* and *NEAT1* (r = 0.43, *p* <0.01). Mouth and genital ulcers were found to be positively and significantly correlated with the BSAS (r = 0.637, *p* < 0.01 and r = 0.556, *p* < 0.01, respectively) and the BDCAF Transformed Index (r = 0.465, *p* < 0.01 and r = 0.372, *p* < 0.01, respectively). Furthermore, cutaneous symptoms, such as Erythema Nodosum, were found to be a significant factor influencing the BDCAF Transformed Index (r = 0.516, *p* < 0.01). The patient’s joint arthralgia was found to be positively and substantially correlated with the BDCAF Transformed Index (r = 0.631, *p* < 0.01). New Eye Involvement (r = 0.389, *p* < 0.01 with BSAS and r = 0.377, *p* < 0.01 with BDCAF) and gastrointestinal symptoms like Diarrhea/BPR were found to positively correlate with BDCAF (r = 0.377, *p* <0.01).

### 2.7. Diagnostic Performance of Serum Biomarkers

**Figure 4:**
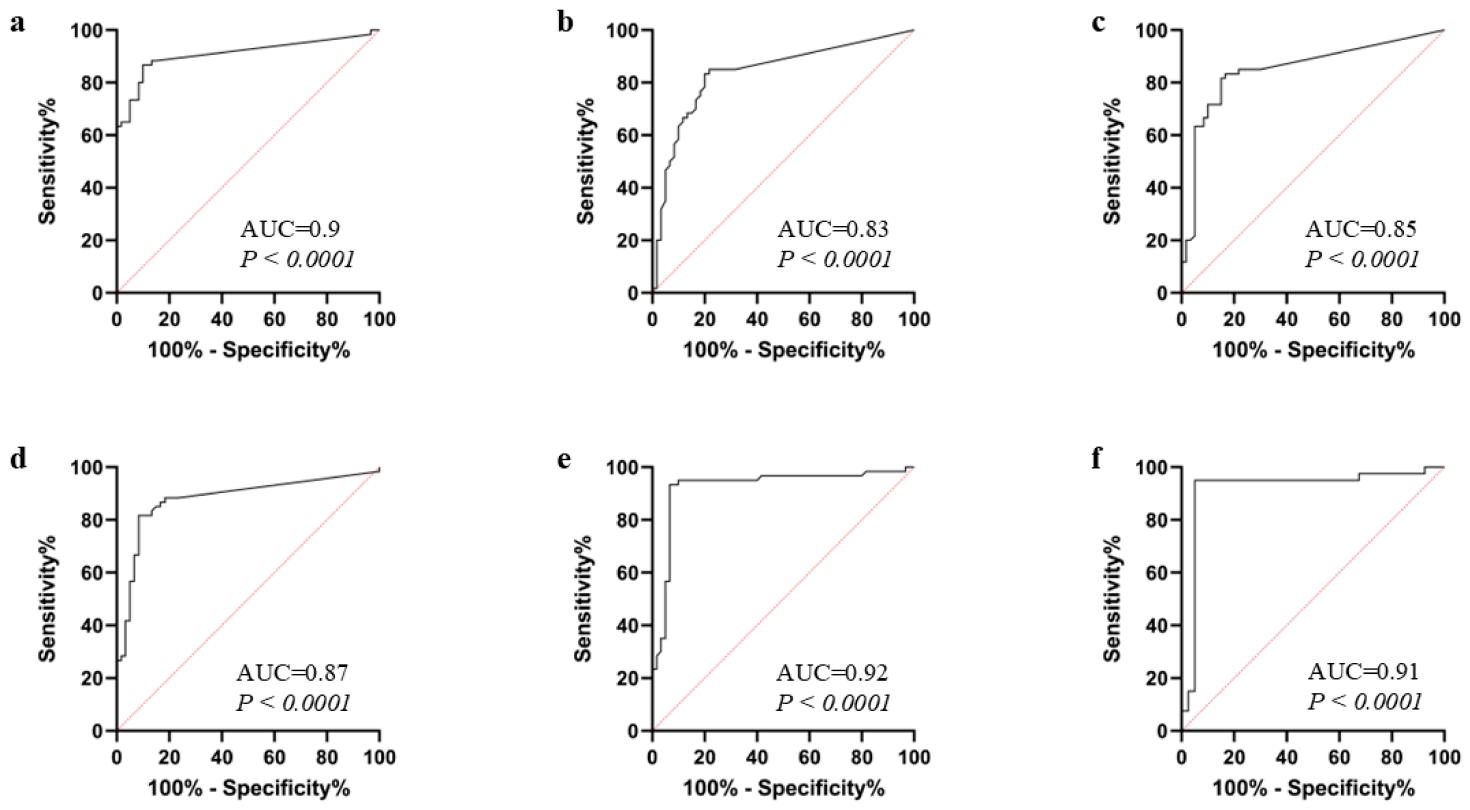
ROC curves of serum biomarkers for the diagnosis of BD. The curves represent the sensitivity (y-axis) versus 100%-specificity (x-axis) for each marker. (**a**) *MEG3* (**b**) *NEAT1* (**c**) *miR-124* (AUC = 0.85); (**d**) *miR-146a*; (**e**) IL-17; (**f**) IL-6. All biomarkers showed a statistically significant discriminatory power (*p* < 0.0001). Abbreviations: AUC, area under the curve.

Receiver operating characteristic (ROC) curve analysis was performed to evaluate the diagnostic efficacy of the studied biomarkers (Table 6 and Figure 4). All studied markers demonstrated highly significant discriminatory power (*p* < 0.0001) in distinguishing BD patients from healthy controls. Among the cytokines, IL-17 demonstrated the most potent diagnostic profile, with the highest area under the receiver operating characteristic curve (AUC) at 0.92 and a sensitivity and specificity of 93.3% at a cut-off value > 59.2. Similarly, IL-6 performed exceptionally well, since its AUC was observed at 0.91, and its sensitivity and specificity were observed at 95%.

Regarding the expression of molecular markers, the long non-coding RNA *MEG3* showed better diagnostic potential, with an AUC of 0.90 and a high specificity of 90% at a cut-off of ≤0.89. *NEAT1* showed promising, diagnostic potential with an AUC of 0.83; the sensitivity and specificity obtained were 83.33% and 79.66%, respectively, at a cut-off of ≤0.75. *miR-146a* and *miR-124* had comparable potential, with the former with an AUC of 0.87, and the latter with an AUC of 0.85. Notably, the cut-off values indicate that elevated levels of cytokines (IL-17, IL-6) and reduced expression levels of the non-coding RNAs (*MEG3*, *NEAT1*, *miR-124*, *miR-146a*) are associated with the disease state.

**Figure 5:**
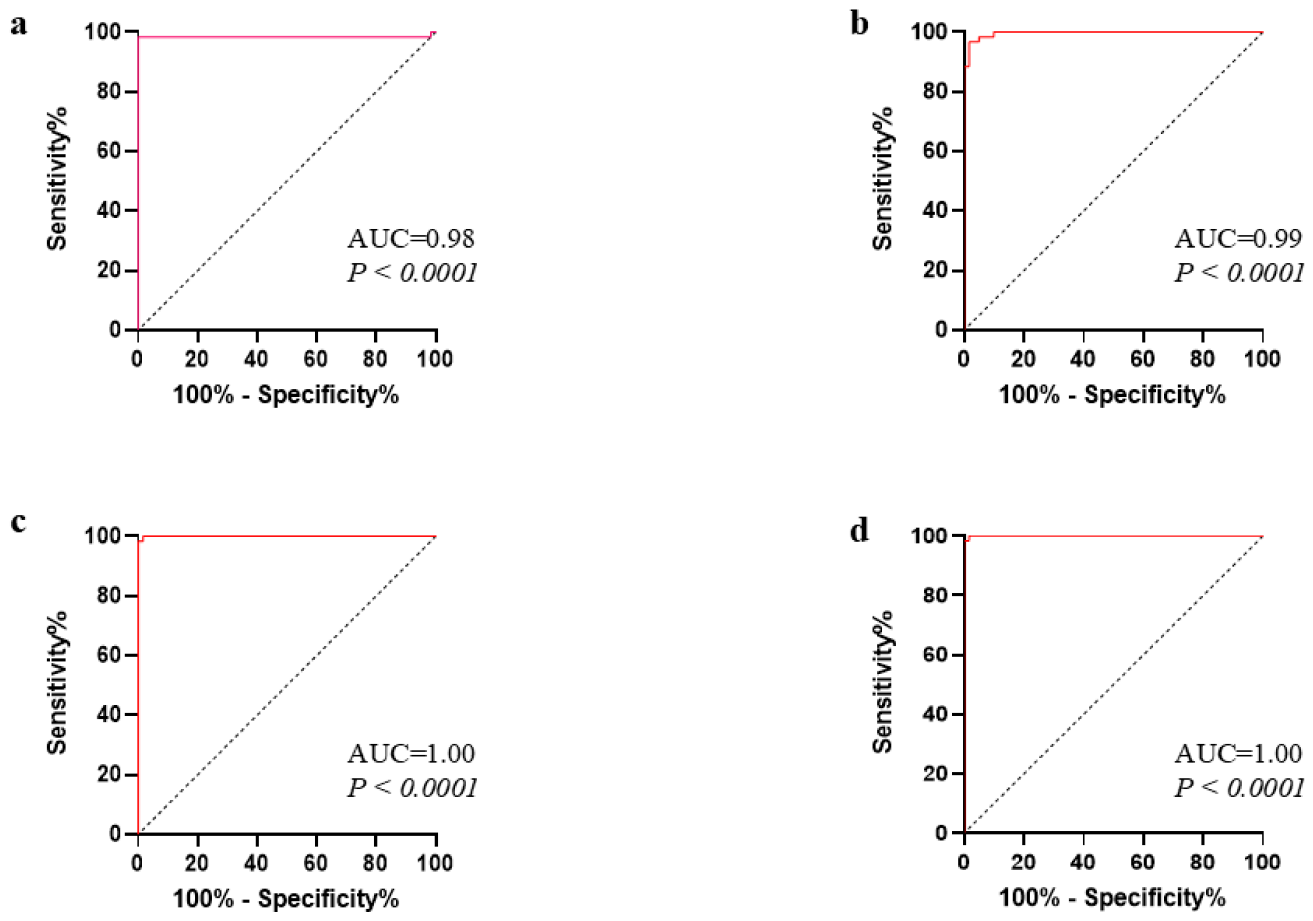
ROC curve analysis of combined diagnostic panels. The Figure shows the results of diagnostic performance for various combinations of markers that were identified by binary logistic regression. Sensitivity (y-axis) versus 100%-specificity (x-axis) for each marker. (**a**) nc MEG 3 combined with nc NEAT1 (AUC = 0.98); (**b**) nc MEG 3 combined with miR-146a; (**c**) nc MEG 3 combined with miR-124; (**d**) miR-146a combined with miR-124 (AUC = 1.00). All combinations exhibited statistically significant discriminatory power (*p* < 0.0001). Abbreviations: AUC, area under the curve; *MEG3*, *Maternally Expressed 3*; *NEAT1*, *Nuclear Paraspeckle Assembly Transcript 1*; *miR*, *microRNA*.

To evaluate the rationale for adopting the multi-marker approach, a combined ROC was performed using binary logistic regression. Probability scores were generated to evaluate the diagnostic value of dual-marker combinations. As shown in Table 6 and Figure 5, the simultaneous use of the *MEG3*/*miR-124* and *miR-124*/*miR-146a* marker sets yielded full diagnostic discrimination ability with an AUC of 1.000 (*p* < 0.0001), with a sensitivity of 100% and specificity of 98.3%. In addition, the combination of the long non-coding RNAs *MEG3* and *NEAT1* recorded a strong predictive quality with an AUC of 0.98, sensitivity of 100% and specificity of 98.3%. On the same note, the *MEG3*/*miR-146a* marker pair had an AUC of 0.99, a sensitivity of 98.3%, and a specificity of 96.6%.

### 2.8. Positivity Rates of Serum Biomarkers

To determine the applicability of the discussed biomarkers in the clinic, the positivity rates for each were calculated using the ROC-derived cut-offs. As presented in Table 7, the test for differences between BD patients and healthy controls identified significant differences for all the studied biomarkers (χ^2^, *p*-value < 0.0001). IL-6 and IL-17 had the highest positivity rates, detecting 95% and 93.3% of BD patients, respectively, with low false-positive rates in the control group of 5% and 6.7%, respectively. *MEG3* was identified as having high potential for discrimination since its levels were found to be low (≤0.89) in 86.7% of BD patients but only 10% of the control group. Similarly, the levels of the two miRNAs, *miR-124* and *miR-146*, showed higher positivity rates in BD patients than in healthy individuals and were statistically validated as having high potential as reliable screening markers for BD.

### 2.9. Multivariate Binary Logistic Regression Analysis

To further verify the diagnostic potential of the aforementioned markers and to exclude the influence of demographics on the results, multivariate binary logistic regression was applied to the markers investigated. Four separate models were established to explore the predictive potential of *MEG3*, *NEAT1*, *miR-124*, and *miR-146a* for BD, adjusting for two variables: Gender and Age. The biomarkers were statistically significant independent predictors of the disease state in all four models (*p* < 0.05), whereas in the *MEG3*, *NEAT1*, and *miR-124* models, neither Age nor Gender was a significant predictor (Table 8).

### 2.10. Machine Learning-Based Diagnostic Validation

**Figure 6:**
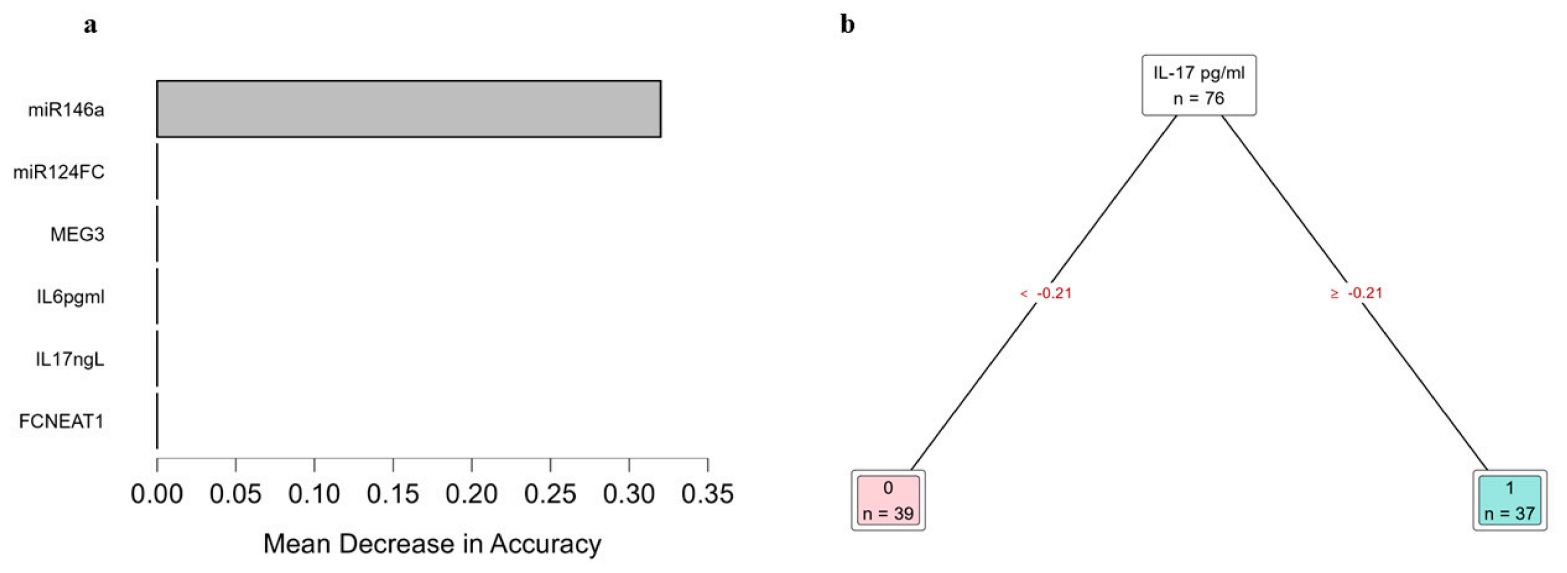
Machine learning analysis of serum biomarkers. (**a**) Random Forest feature-importance plot showing miR-146a as the most informative individual feature based on the mean decrease in accuracy. (**b**) Decision Tree model identifying serum IL-17 as the root node, indicating that IL-17 provided the most efficient initial split between patients with Behçet’s disease and healthy controls in the final tree model. The Decision Tree was generated using a training set of 76 subjects (63% of the cohort), whereas the remaining 44 subjects were reserved for validation and testing. Consequently, the total number of subjects displayed in the tree diagram is 76 rather than 120.

To complement the conventional statistical analyses, machine learning models were employed to further evaluate the diagnostic contribution of the investigated biomarkers. Using the Random Forest algorithm, biomarkers were ranked according to their contribution to the overall predictive performance of the ensemble model. As shown in Figure 6a, *miR-146a* demonstrated the highest feature importance, with the greatest mean decrease in accuracy (0.320), indicating that it was the most informative individual variable for distinguishing patients with Behçet’s disease from healthy controls within the Random Forest model.

A Decision Tree model was subsequently constructed to develop an interpretable classification algorithm. As illustrated in Figure 6b, serum IL-17 was selected as the root node because it provided the most efficient initial split between the two study groups. This finding should not be interpreted as indicating that IL-17 is the only relevant classifier or that *miR-146a* is unnecessary for disease classification. Rather, the two machine learning approaches provide complementary information. The Random Forest model evaluates the overall contribution of each variable across an ensemble of decision trees, whereas the Decision Tree identifies the single variable that produces the optimal first partition within one simplified classification model. Therefore, the identification of *miR-146a* as the most important feature in the Random Forest analysis and IL-17 as the root node in the Decision Tree are not contradictory, but instead reflect different analytical perspectives of the same biomarker signature.

### 2.11. In Silico Explanation of the NEAT1–miRNA–Cytokine Regulatory Axis

To explore the potential molecular interactions underlying the observed ncRNA dysregulation, the same bioinformatic workflow was initially applied to both *NEAT1* and *MEG3* using the ENCORI (StarBase v2.0) (http://starbase.sysu.edu.cn/) (accessed on 5 January 2026) and miRTarBase databases (https://mirtarbase.cuhk.edu.cn/) (accessed on 5 January 2026). However, only *NEAT1* demonstrated predicted interactions involving the investigated microRNAs (*miR-124-3p* and *miR-146a-5p*) that fulfilled the predefined selection criteria for subsequent regulatory network construction. Consequently, only the *NEAT1*-associated regulatory network was included in the downstream bioinformatic analyses and graphical representation.

**Figure 7:**
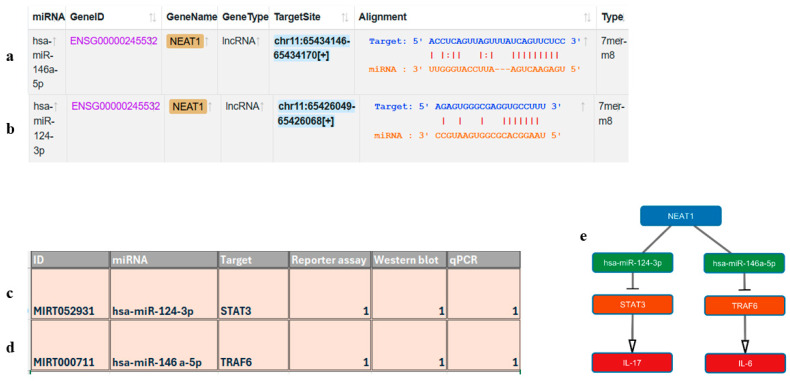
Integrated bioinformatic analysis and construction of a putative ncRNA regulatory network associated with Behçet’s disease. (**a**,**b**) In silico prediction of potential binding sites between lncRNA *NEAT1* and *miR-146a-5p* (**a**) and *miR-124-3p* (**b**) using the ENCORI database. These analyses identified predicted lncRNA–miRNA binding sites suggesting potential interactions between NEAT1 and the investigated miRNAs. However, these bioinformatic predictions were not experimentally validated in the present study. (**c**,**d**) Experimentally validated downstream targets retrieved from the miRTarBase database. STAT3 was identified as a validated target of miR-124-3p (**c**), whereas TRAF6 was identified as a validated target of miR-146a-5p (**d**), based on experimentally validated interactions curated in miRTarBase. (**e**) Integrated regulatory network constructed using Cytoscape by combining the experimentally observed expression profiles with bioinformatically predicted lncRNA–miRNA interactions and experimentally validated miRNA–target relationships. The network summarizes the coordinated dysregulation of *NEAT1*, *MEG3*, *miR-124-3p*, and *miR-146a-5p* observed in patients with Behçet’s disease, together with increased circulating IL-17 and IL-6 levels. The connecting edges between *NEAT1* and the investigated miRNAs represent bioinformatically predicted interactions retrieved from the ENCORI database and are included to visualize the predicted interaction network. They should not be interpreted as indicating the direction of regulation or as evidence of a direct causal ceRNA mechanism in the current study. In contrast, the interactions between miR-124-3p and STAT3, and between miR-146a-5p and TRAF6, are supported by experimentally validated evidence retrieved from miRTarBase. Collectively, the Cytoscape network integrates our experimental findings with publicly available bioinformatic and experimentally validated data, providing a hypothesis-generating framework for future mechanistic studies rather than a confirmed molecular pathway.

First, alignment analysis identified specific, conserved binding sites for *miR-146a* and *miR-124* within the lncRNA *NEAT1* sequence (Figure 7a,b). Second, downstream target prediction was functioned to link these ncRNAs with the inflammatory phenotype. Target validation evaluation proved that *miR-146a* targets the 3’UTR of TRAF6 (Figure 7c), an important upstream adaptor of the NF-κB signaling pathway [23]. In addition, *miR-124* was shown to target STAT3 (Figure 7d), the master transcription factor of Th17 cell differentiation [24,25]. Based on these validated interactions, a hierarchical regulatory network was developed in Cytoscape 3.10.1 (Figure 7e).

## 3. Discussion

Behçet’s disease, which encompasses most body systems, is an intricate multisystemic disease causing recurrent inflammation and immune system disorders [2]. Even though this disease comprises considerable morbidity, the molecular mechanisms underlying the pathogenesis of BD, as well as the association between non-coding RNAs and pro-inflammatory cytokines, have not yet been entirely elucidated [26]. The present study identified a molecular signature for patients with BD, characterized by the simultaneous repression of ncRNAs, such as *MEG3* and *NEAT1*, and *miR-124* and *miR-146a*. Conversely, Th17-associated pro-inflammatory cytokines, including IL-17 and IL-6, were significantly upregulated. The identified biomarker panel also demonstrated promising discriminatory performance within the present cohort, which was further supported by machine learning-based predictive modeling. The machine learning findings should be interpreted in a model-specific manner. Random Forest feature importance reflects the contribution of each variable to the overall predictive performance of an ensemble of decision trees, whereas the Decision Tree root node identifies the single variable that provides the most efficient initial partition of the study population. Consequently, the identification of IL-17 as the root node in the Decision Tree does not imply that *miR-146a* is unnecessary for disease classification. Rather, *miR-146a* demonstrated the highest feature importance in the Random Forest analysis, supporting its substantial contribution to the overall molecular classification signature, while IL-17 served as the most informative first-level inflammatory stratifier within the simplified Decision Tree model. These complementary findings highlight distinct but interconnected aspects of the diagnostic landscape in Behçet’s disease.

Recent high-throughput transcriptomic studies have identified mRNA-based inflammatory signatures in Behçet’s disease characterized by dysregulation of innate immune responses, neutrophil activation, interferon signaling, NF-κB activation, and Th17-associated pathways [27,28]. While these studies identify downstream effector genes, the circulating ncRNA–cytokine signature described in the current study may represent an upstream regulatory layer that contributes to the modulation of these inflammatory networks. Unlike mRNAs, lncRNAs and miRNAs participate in epigenetic and post-transcriptional regulation and generally exhibit greater stability in circulation, making them attractive minimally invasive biomarkers [28,29]. Accordingly, the present ncRNA panel should be considered complementary to existing transcriptomic signatures rather than a replacement. Mechanistically, *miR-124* has been shown to regulate STAT3 signaling, whereas *miR-146a* negatively regulates the TRAF6/NF-κB pathway [30,31] (Taganov et al., 2006; Qin, Z., 2017), providing biologically plausible links between the ncRNA alterations observed in the present study and inflammatory mRNA signatures previously described in Behçet’s disease. Future multi-omics studies integrating circulating ncRNAs, mRNA transcriptomics, cytokine profiling, and functional validation will be necessary to determine whether combined biomarker panels improve disease diagnosis, patient stratification, and mechanistic understanding.

The predominance of male patients observed in the present cohort is consistent with the epidemiological characteristics of Behçet’s disease reported in countries along the historical Silk Road, including the Middle East and Mediterranean regions, where the disease is generally more prevalent in men and tends to exhibit a more severe clinical course, particularly with ocular and vascular involvement [32,33,34]. In contrast, cohorts from Northern Europe and North America often demonstrate a more balanced sex distribution and less pronounced sex-related differences in disease severity [32,34]. This sex distribution is also comparable to several previously published Egyptian Behçet’s disease cohorts, in which male patients represented the majority of the study population [35,36]. Importantly, the control group was matched for sex, no significant differences in the investigated biomarkers were identified between male and female participants within our cohort, and sex was included as a covariate in the multivariable logistic regression analysis to minimize potential confounding. Nevertheless, although the observed sex distribution reflects the regional epidemiology of Behçet’s disease, the predominance of male patients may have reduced the statistical power to detect subtle sex-specific differences in ncRNA expression. Therefore, larger multicenter studies including more balanced sex distributions are warranted to determine whether sex influences the molecular profile or diagnostic performance of these biomarkers in Behçet’s disease.

One of the principal findings of the current study was the marked downregulation of *MEG3* in the serum of patients with Behçet’s disease. *MEG3* is a well-characterized long non-coding RNA implicated in immune regulation and inflammatory homeostasis. In the present cohort, serum *MEG3* levels were inversely correlated with IL-17 levels (r = −0.42), suggesting a potential association between reduced *MEG3* expression and enhanced Th17-associated inflammation. Previous experimental studies have demonstrated that *MEG3* can negatively regulate STAT3 signaling, a key pathway involved in Th17 differentiation and cytokine production. Although this investigation does not establish a causal relationship, our findings are consistent with the hypothesis that reduced *MEG3* expression may contribute to diminished restraint of STAT3 signaling, thereby favoring Th17 cell expansion and increased production of IL-17 and IL-6 in Behçet’s disease.

Likewise, a significant downregulation of *NEAT1* was observed in patients with Behçet’s disease. Although *NEAT1* is frequently reported to be upregulated in acute inflammatory and viral conditions [37,38], the present findings suggest that its expression may be differentially regulated in chronic autoimmune diseases. Previous studies have also implicated *NEAT1* in regulating several inflammatory pathways, including inflammasome activation, in different immune-mediated disorders. However, whether similar mechanisms operate in Behçet’s disease remains unknown and was not investigated in the present study. Our multivariate logistic regression analysis further identified reduced *NEAT1* expression as an independent predictor of Behçet’s disease after adjustment for age and sex. In addition, the positive correlation between *MEG3* and *NEAT1* (r = 0.43) raises the possibility that these two lncRNAs may be coordinately regulated through shared upstream transcriptional or epigenetic mechanisms, although this hypothesis requires further experimental investigation.

Interestingly, previous studies investigating *NEAT1* expression in BD have produced conflicting findings. Hussein et al. similarly reported significant downregulation of serum *NEAT1* in Egyptian BD patients together with elevated IL-17 levels, supporting the current observations. In contrast, Mohammed et al. demonstrated increased *NEAT1* expression and identified positive associations with disease activity and multiple organ manifestations [36]. These divergent findings may indicate that *NEAT1* behaves as a context-dependent inflammatory regulator whose expression profile varies according to disease stage, inflammatory burden, vascular involvement, therapeutic exposure, or tissue-specific immune responses. Such variability has also been described for several immune-regulatory lncRNAs in other autoimmune diseases, emphasizing the dynamic nature of non-coding RNA signaling during chronic inflammation [35,36].

The positive correlation between *MEG3* and *NEAT1* suggests the possibility of shared upstream regulatory mechanisms [39]. In autoimmune diseases, coordinated dysregulation of lncRNAs has been linked to epigenetic alterations including DNA methylation and histone modifications [40,41]. Although epigenetic mechanisms were not investigated in the current study, these observations provide a biologically plausible explanation for the coordinated depletion of both lncRNAs observed in BD [42].

The observed downregulation of *miR-124* and *miR-146a* further supports the presence of a dysregulated inflammatory ncRNA network in Behçet’s disease. *miR-146a* is a well-established negative regulator of innate immune responses through modulation of the TRAF6/NF-κB signaling pathway. Therefore, reduced *miR-146a* expression may weaken an important negative feedback mechanism that normally limits excessive inflammatory signaling. Consistent with this observation, the Random Forest model ranked *miR-146a* as the most informative individual feature for distinguishing patients with Behçet’s disease from healthy controls, highlighting its potential contribution to the molecular signature identified in the present cohort.

The pronounced reduction in *miR-146a* observed in the current cohort is consistent with previous reports highlighting the anti-inflammatory role of this microRNA in BD. El Khateeb et al. and Ahmed et al. independently reported significant downregulation of circulating *miR-146a* in Egyptian BD patients, together with promising diagnostic performance and associations with inflammatory activity. Biologically, *miR-146a* functions as an important negative regulator of innate immune activation through modulation of TRAF6/NF-κB signaling. Therefore, depletion of *miR-146a* may weaken one of the major intracellular feedback mechanisms responsible for limiting excessive cytokine production, thereby facilitating the persistent inflammatory activation observed in BD. The current findings further support the concept that *miR-146a* dysregulation represents a central molecular component of BD-associated immune imbalance rather than a secondary epiphenomenon of inflammation alone [43,44].

Nevertheless, the literature regarding *miR-146a* expression in BD remains somewhat heterogeneous. Ibrahim et al. reported elevated rather than reduced circulating *miR-146a* levels in BD patients and demonstrated associations with ocular and vascular manifestations. Such discrepancies across studies are not unexpected in complex immune-mediated diseases and may reflect differences in disease activity status, treatment exposure, ethnicity, sample processing protocols, normalization strategies, or organ-dominant clinical phenotypes. It is therefore plausible that *miR-146a* expression undergoes dynamic fluctuations during different inflammatory stages of BD, functioning as part of a compensatory regulatory response in some disease settings while becoming exhausted or suppressed in others [45].

Bioinformatic analyses identified putative interactions between *NEAT1* and both *miR-124* and *miR-146a*, suggesting a potential lncRNA–miRNA regulatory relationship. Previous experimental studies have demonstrated *NEAT1*–*miR-124* and *NEAT1*–*miR-146a* interactions in other biological contexts using luciferase reporter assays, RNA immunoprecipitation, RNA pull-down, and functional rescue experiments [39,40,41,42]. However, these interactions were not functionally validated in the current investigation. Importantly, the concurrent downregulation of *NEAT1*, *miR-124*, and *miR-146a* observed in the present cohort should not be interpreted as evidence of a canonical ceRNA- or miRNA-sponge mechanism in Behçet’s disease. In such a model, reduced NEAT1 abundance would be expected to increase miRNA availability rather than explain reduced miRNA expression. Instead, the parallel decrease in *NEAT1* and the investigated miRNAs may reflect shared upstream inflammatory or epigenetic regulation, altered RNA processing or stability, changes in extracellular RNA release, or differences in the cellular sources contributing to the circulating RNA pool. These possibilities remain speculative and require direct functional validation in future studies. Although *MEG3* exhibited strong diagnostic performance and emerged as an independent predictor of Behçet’s disease, the same bioinformatic workflow did not identify predicted *MEG3*–miRNA interactions that satisfied the predefined selection criteria. Consequently, *MEG3* was not incorporated into the proposed *NEAT1*-centered regulatory network and may contribute to Behçet’s disease through alternative molecular mechanisms that warrant further investigation.

Downstream of the investigated miRNAs, previous experimental studies suggest functional cross-talk between the STAT3 and NF-κB signaling pathways. Under physiological conditions, these pathways are tightly regulated through reciprocal interactions to maintain immune homeostasis and prevent excessive inflammation [46,47,48]. Reduced *miR-124* expression has been shown to enhance STAT3 signaling [49,50], whereas reduced *miR-146a* expression may augment TRAF6/NF-κB signaling [41]. Consequently, reciprocal activation of these pathways may promote IL-6 production, further amplifying JAK/STAT3 signaling and favoring Th17-cell differentiation while limiting regulatory T-cell responses [49,51,52,53,54]. Collectively, these experimentally supported mechanisms provide a biologically plausible framework for interpreting the present findings and may help explain the enhanced IL-6/IL-17 inflammatory profile observed in Behçet’s disease. However, these intracellular signaling events were not directly investigated in the present research and therefore require future functional validation.

The marked elevation of IL-17 observed in the present study is strongly aligned with the established Th17-centered inflammatory model of Behçet’s disease. A recent meta-analysis involving more than 900 BD patients confirmed significantly higher circulating IL-17 levels in BD compared with healthy controls, reinforcing the reproducibility of this cytokine abnormality across different populations. In parallel, accumulating evidence indicates that IL-6 serves as a critical upstream driver of Th17 polarization and amplification in BD. Experimental studies have additionally shown that dysregulated non-coding RNAs can directly influence IL-6 expression, providing a mechanistic bridge between epigenetic dysregulation and cytokine-mediated inflammation. Collectively, these observations support the concept that the ncRNA alterations identified in the present cohort may actively participate in sustaining the IL-6/IL-17 inflammatory axis rather than simply reflecting downstream inflammatory damage [55,56].

Interestingly, *miR-124* showed distinct associations with neurological and ocular manifestations, suggesting that its biological role may differ according to organ involvement. Although the observed correlations were derived from the present cohort, the possible biological explanation is supported by previous experimental and clinical studies demonstrating that *miR-124* is highly enriched in the central nervous system and plays important roles in regulating neuro-inflammation, microglial activation, and central nervous system immune homeostasis [57,58]. Altered *miR-124* expression has been implicated in several neuro-inflammatory disorders, supporting the possibility that tissue-specific regulatory mechanisms may influence its expression and function in Behçet’s disease [57,58]. However, these subgroup analyses should be interpreted cautiously because of the relatively limited number of patients with specific clinical manifestations and require validation in larger independent cohorts.

In addition, the data reveals that the studied markers are associated with specific organ involvement. The data shows a significant association between *MEG3* and ocular involvement. In patients with active ocular involvement, serum *MEG3* levels were significantly lower than in those without ocular involvement (*p* = 0.01). Reduced *MEG3* expression may contribute to enhanced Th17-associated inflammatory responses. These findings suggest that *MEG3* may have potential as a biomarker of ocular involvement, although prospective validation is required. Moreover, it was found that *NEAT1* was significantly downregulated in patients with major vessel involvement (*p* = 0.03). The vascular inflammation seen in BD is due to endothelial dysfunction [59]. Since endothelial integrity is maintained by *NEAT1* [60,61], its suppression in patients may reflect loss of *NEAT1* at the vascular level.

Recent evidence further supports the relevance of *MEG3* dysregulation in Behçet’s disease. Abobakr et al. demonstrated significant serum downregulation of *MEG3* in BD patients and reported a strong diagnostic performance for this lncRNA. Interestingly, they also observed lower *MEG3* expression in patients with ocular involvement, which closely parallels the present findings linking reduced *MEG3* levels with active eye manifestations. This concordance across independent cohorts strengthens the possibility that *MEG3* depletion is not merely a nonspecific inflammatory event, but may reflect pathogenic mechanisms associated with immune-mediated ocular inflammation in BD. Given the established role of *MEG3* in regulating inflammatory and STAT3-related signaling pathways, its reduced expression may contribute to the exaggerated Th17-driven responses characteristic of the disease [62].

This specific association between *NEAT1* downregulation and major vessel involvement (*p* = 0.03) provides compelling evidence for the protective role of non-coding RNAs in the vascular endothelium [63]. Recent functional assays in vascular biology have demonstrated that *NEAT1* regulates endothelial cell migration, proliferation, and sprout formation by modulating the expression of Kruppel-like Factor 2 (KLF2) and endothelial nitric oxide synthase (eNOS) [64,65]. When *NEAT1* expression is suppressed, as seen in our BD cohort, this protective endothelial shield is lost. Collectively, these mechanisms may help explain the pronounced vascular inflammation and thrombotic tendency observed in Angio-BD [59,66,67,68].

A distinction was noted between the diagnostic and activity markers. A positive significant correlation was noted between the levels of serum IL-17 and the BDCAF Patient Index Score (r = 0.274; *p* = 0.03). Furthermore, significant correlations were noted for the levels of IL-17 with both joint arthritis (r = 0.358) and major vessel involvement (r = 0.276), supporting its potential involvement in inflammatory activity during active disease and could be used as an indication of flare-ups of the disease process. In contrast, the expression levels of *MEG3*, *NEAT1*, *miR-124*, and *miR-146a* did not correlate with the BDCAF and BSASs (*p* > 0.05). These findings may indicate that their dysregulation reflects a relatively stable molecular characteristic of BD rather than a purely activity-dependent alteration.

According to diagnostic performance, the markers performed well in differentiating the BD group from the healthy control group, with AUCs ranging from 0.83 to 0.92. The results indicated that the dual marker combinations, such as *MEG3* and *NEAT1* and *miR-124* and *miR-146a*, achieved near-perfect classification (AUC = 1.00). Additional evaluation using machine learning approaches further supported the robustness of the observed biomarker signature within the present cohort. The Decision Tree analysis identified serum IL-17 as the top stratification node, which segregated most patients (*n* = 76 training set) with high precision. Machine learning analyses further supported the discriminatory performance of the identified biomarker signature within the present cohort.

From a clinical perspective, this molecular profile provides a dual utility model for precision medicine. The long non-coding RNAs *MEG3* and *NEAT1* are strong and stable biomarkers, providing high diagnostic accuracy (AUC = 1.000 for combined panels). On the other hand, IL-17 is a dynamic “state” marker that strongly correlates with active arthritis and disease flare-ups, making it a strong candidate for monitoring treatment response. These results not only reveal the epigenetic mechanisms of the disease process in BD but also point to the *NEAT1*-miRNA-STAT3/TRAF6 pathway as a potential target for the development of new RNA-based therapies.

Although the combined biomarker panels demonstrated exceptionally high discriminatory performance within the present cohort, these findings should be interpreted with appropriate caution. Extremely high AUC values may partially reflect the controlled case–control design and the use of healthy controls rather than clinically overlapping inflammatory diseases. In addition, the relatively limited sample size increases the possibility of model overfitting, particularly in machine learning-based analyses. Therefore, larger multicenter studies incorporating independent validation cohorts and disease-control populations are necessary before these biomarker panels can be considered for routine clinical application. Nevertheless, the consistent performance observed across both conventional statistical and machine learning approaches supports the biological relevance of the identified ncRNA-inflammatory signature in BD.

Although the investigated biomarker panel demonstrated excellent discrimination between patients with Behçet’s disease and healthy controls, the present case–control design does not permit conclusions regarding disease specificity because no disease-control group was included. Similar alterations in inflammatory cytokines and non-coding RNAs have been reported in other autoimmune and inflammatory disorders. Therefore, the present findings should be interpreted as demonstrating diagnostic discrimination against healthy individuals rather than disease-specific molecular signatures. Future studies including clinically relevant disease-control cohorts will be essential to determine the specificity and potential clinical utility of these biomarkers in the differential diagnosis of Behçet’s disease.

### Limitations

Several limitations of the current research should be acknowledged. First, the relatively limited sample size and single-center design may restrict the generalizability of the findings to broader Behçet’s disease populations. Second, a major limitation of the present study is that the diagnostic analyses were performed exclusively against healthy controls without inclusion of disease-control groups. Consequently, although the investigated biomarkers demonstrated excellent discrimination between Behçet’s disease patients and healthy individuals, their specificity for Behçet’s disease cannot be established because similar molecular alterations may occur in other inflammatory or autoimmune disorders. Validation in independent cohorts including clinically relevant disease-control populations will therefore be essential before these biomarkers can be considered disease-specific diagnostic candidates. Third, although bioinformatic analyses and correlation patterns supported the proposed ncRNA-associated regulatory network, no functional validation experiments were performed to confirm the predicted molecular interactions. Fourth, all molecular analyses were performed using serum samples; therefore, tissue-specific regulatory mechanisms within affected organs could not be directly investigated. Fifth, the cross-sectional design precluded assessment of temporal changes in biomarker expression across different disease stages, treatment responses, and long-term clinical outcomes. Finally, although the predominance of male patients reflects the epidemiological characteristics of Behçet’s disease in our geographic region, the relatively unbalanced sex distribution may have reduced the statistical power to detect subtle sex-specific differences in ncRNA and cytokine expression. Consequently, validation in larger multicenter cohorts with more balanced sex representation will be important to confirm the generalizability of the present findings.

Therefore, larger multicenter prospective studies incorporating balanced demographic characteristics, disease-control cohorts, longitudinal follow-up, and mechanistic validation experiments are warranted to further establish the biological significance, disease specificity, and clinical utility of the identified ncRNA-associated inflammatory signature in Behçet’s disease.

## 4. Materials and Methods

### 4.1. Study Population

This observational, analytical case–control study was conducted at the outpatient clinics of the Rheumatology and Rehabilitation Department, Fayoum University Hospitals. The study included 60 patients with Behçet’s disease (BD) diagnosed according to the International Criteria for Behçet’s Disease (ICBD) [69], and 60 age- and sex-matched healthy controls recruited from blood bank volunteers.

Participants aged ≥18 years were eligible. Exclusion criteria included pregnancy, presence of other autoimmune or inflammatory diseases, other forms of vasculitis, malignancy, and known hypercoagulable disorders. All participants underwent detailed clinical evaluation, including medical history, physical examination, and laboratory investigations. Disease activity was assessed using the Behçet’s Disease Current Activity Form (BDCAF) and Behçet’s Syndrome Activity Score (BSAS) [70,71].

The study was conducted in accordance with the Declaration of Helsinki and approved by the Ethics Committee of the Faculty of Medicine, Fayoum University (Approval No. R831-2026). Written informed consent was obtained from all participants.

### 4.2. Blood Specimen Collection

Venous blood samples (5 mL) were collected from each participant under standardized conditions. Samples were allowed to clot at room temperature for 30 min, followed by centrifugation at 4000× *g* for 15 min. The separated serum was aliquoted and stored at −80 °C until further analysis.

### 4.3. Biomarker Selection

The selected molecular panel was designed to investigate a putative epigenetic–inflammatory regulatory axis in Behçet’s disease. The long non-coding RNAs *NEAT1* and *MEG3* were chosen based on their reported roles in immune regulation, endothelial function, and inflammatory signaling pathways [72]. The microRNAs *miR-124* and *miR-146a* were included due to their well-established anti-inflammatory functions. Specifically, *miR-124* is known to negatively regulate the STAT3 signaling pathway, a key driver of Th17 cell differentiation, while *miR-146a* functions as a negative feedback regulator of the NF-κB pathway through targeting TRAF6 [73,74]. To reflect downstream inflammatory activity, the pro-inflammatory cytokines IL-17 and IL-6 were quantified as functional readouts of Th17-mediated immune responses. Collectively, this panel was selected to capture different regulatory layers of the proposed lncRNA–miRNA–cytokine axis.

### 4.4. RNA Extraction and Reverse Transcription

According to the manufacturer’s instructions, we utilized a miRNeasy extraction kit (QIAGEN, Hilden, Germany; Cat. No. 217004) to extract total RNA from the serum. It is important to note that a separate miRNA-enriched fraction was not extracted; rather, the standard protocol was followed to co-purify total RNA, which efficiently captures both large RNAs (including the lncRNAs *MEG3* and *NEAT1*) and small RNAs (including *miR-124* and *miR-146a*) within a single eluate. This ensured all subsequent analyses were performed on the same RNA pool. Next, a NanoDrop™ 2000 Spectrophotometer (Thermo Fisher Scientific, Wilmington, DE, USA) was used at 260/280 nm to check the RNA concentration and purity. A ratio greater than 1.8 meant that the RNA yield and purity were satisfactory.

Then, to get cDNA for *MEG3*, *NEAT1*, and GAPDH the following supplier’s instructions were followed, a standardized amount of 20 ng of total RNA was reverse-transcribed with a RevertAid First Strand cDNA Synthesis kit (Thermo Fisher Scientific, USA; Cat. No. K1622). The reactions were put in a thermal cycler that was set to 25 °C for 5 min for primer annealing, 42 °C for 60 min for processing and then 70 °C for 5 min for termination [75]. After that, the cDNA was kept at −20 °C for further use.

### 4.5. Quantitative Real-Time Polymerase Chain Reaction (qPCR)

Quantitative real-time polymerase chain reaction (qPCR) was performed to quantify the expression levels of the selected lncRNAs. The resulting cDNA (2 µL per reaction) was utilized as the input template for quantitative real-time PCR (qPCR). All samples were tested in technical duplicates to ensure reliability and reproducibility. This was done using specific primers from Thermo-Fisher Scientific in Germany and Maxima SYBR Green qPCR Master Mix (Thermo-Fischer Scientific, USA, Cat. No. K0222) as indicated by the manufacturer [76]. Table 9 lists the sequences of the primers used in the PCR. The parameters for cycling were as follows: 10 min at 95 °C, 40 cycles at 95 °C for 15 s, and 60 °C for 60 s. *Glyceraldehyde 3-phosphate dehydrogenase* (*GAPDH*) served as the housekeeping reference gene for *NEAT1* and *MEG3* [77]. *GAPDH* was selected as the endogenous reference gene based on its previous use in circulating lncRNA studies and was used consistently for normalization throughout the current investigation. We acknowledge that no universally accepted endogenous reference gene has yet been established for serum-derived lncRNA quantification, and normalization strategies for circulating ncRNAs remain an evolving methodological consideration [78,79].

For the quantification of microRNAs, 20 ng of total RNA was reverse-transcribed into cDNA using the miScript II RT Kit (Cat. No. 218161; QIAGEN, Hilden, Germany) according to the manufacturer’s protocol. The resulting cDNA (2 µL per reaction) was utilized as the input template for quantitative real-time PCR (qPCR) employing the miScript SYBR Green PCR Kit (Cat. No. 218073; QIAGEN). The reaction mixtures (20 µL total volume) were formulated using the miScript Universal Primer as the reverse primer, alongside target-specific miScript Primers (forward primers) designed to detect *miR-146a*, *miR-124*, and the endogenous reference miRNA SNORD68 [80]. All qPCR assays were performed on a Rotor-Gene Q instrument (Qiagen). The thermal cycling conditions consisted of an initial activation step at 95 °C for 30 s, followed by 40 cycles of denaturation at 94 °C for 15 s, annealing at 55 °C for 30 s, and extension at 70 °C for 30 s. Post-amplification melting curve analysis was conducted to confirm the specificity of the PCR products. Subsequently, the relative expression levels of the selected RNAs in each sample were determined via the 2^−ΔΔCt^ method [81].

### 4.6. Quantification of Serum IL-6 and IL-17

Frozen serum samples were thawed on ice for analysis. The quantitative measurement of IL-6 and IL-17 concentration was carried out by the ELISA method by using commercial Sandwich ELISA kits (Ray Biotech, Norcross, GA, USA; Cat. No. [ELH-IL-6-CL] and [ELH-IL17]), strictly following the instructions given by the manufacturer. In brief, 50 µL of standard and diluted serum samples were added to 96-well plates pre-coated with specific capture antibodies. After incubation and appropriate washing steps, a biotinylated detection antibody and Streptavidin-HRP solution were added one after another. The reaction was developed with a TMB one-step substrate reagent and stopped with a stop solution. The optical density was measured instantly at 450 nm with a microplate reader.

### 4.7. Bioinformatics Analysis and Network Construction

To elucidate the mechanistic framework underlying the “*NEAT1*−miRNA−Cytokine” axis, a comprehensive, two-step bioinformatics analysis was performed, clearly distinguishing between predictive in silico modeling and database-retrieved experimental validation.

a. Exclusively In Silico Predictions (lncRNA–miRNA interactions): The interactions between the lncRNA *NEAT1* and the microRNAs of interest (*miR-124-3p* and *miR-146a-5p*) were exclusively in silico predictions. We utilized the ENCORI (StarBase v2.0) platform (http://starbase.sysu.edu.cn/), a stringent database for decoding pan-cancer and disease-related ncRNA networks. Alignment analysis was conducted to identify specific, conserved complementary binding sequences (seed regions) between *NEAT1* and both microRNAs, serving as the theoretical basis for the proposed “molecular scaffold” mechanism.

b. Experimentally Validated Targets (miRNA–mRNA interactions): To link these microRNAs to the observed inflammatory phenotype (IL-17 and IL-6 elevation), downstream target analysis was conducted using miRTarBase (https://mirtarbase.cuhk.edu.cn/). Unlike predictive algorithms, miRTarBase strictly curates microRNA–target interactions (MTIs) that possess strong experimental validation from the published literature. We retrieved MTIs confirming that *miR-124* specifically targets the 3′UTR of STAT3 (the master regulator of Th17 differentiation) and *miR-146a* targets the 3′UTR of TRAF6 (the upstream adaptor of the NF-κB pathway). The database confirmed that these specific interactions have been previously validated via robust experimental techniques, including luciferase reporter assays, Western blotting, and qPCR.

Finally, based on these integrated in silico predictions and experimentally validated targets, a hierarchical regulatory network (*NEAT1* → *miR-124*/*miR-146a* → STAT3/TRAF6) was constructed and mapped using Cytoscape software (version 3.10.1) for visual representation.

### 4.8. Statistical Analysis

Data were analyzed using the Statistical Package for the Social Sciences (SPSS.22), developed by IBM, Armonk, NY, USA, and Prism 9.5.1 statistics tool, USA. In this study, the data of categorical variables were expressed as numbers and percentages, while the numerical data were expressed as mean ± SEM, median (25–75% percentiles), or range, where appropriate. D’Agostino and Pearson, Kolmogorov–Smirnov, and Shapiro–Wilk analyses were conducted to evaluate the normality of data distribution. The following statistical methods were conducted for comparisons of numerical variables, where applicable: Mann–Whitney U test, *t*-test, Kruskal–Wallis followed by Dunn’s, or one-way ANOVA followed by Tukey’s or post hoc test. Fisher’s exact analysis was conducted to compare categorical data within the study. For evaluating the discriminatory capability of the markers, the receiver operating characteristic (ROC) test was applied, and its area under the curve (AUC) was calculated subsequently. The AUCs were placed into three groups, AUCs ranging from 0.6 to 0.7, from 0.7 to 0.9, and above 0.9, for defining the biomarker as a significant, promising, and outstanding discriminant, respectively. In this research, logistic regression analysis, both univariate and multivariate, has been applied for categorizing the predictor markers for Behçet’s cases compared with the control group. In this study, a stepwise-forward multivariate logistic regression test has been implemented to acquire the final markers associated with the result of being identified for the possibility of obtaining a diagnosis for BD. For calculating correlations for measurement data, Spearman’s rho correlation coefficient test has been adopted. In these statistical calculations, statistical significances for results have been perceived when the *p*-values for any test were found to be less than 0.05 in their two-tailed value.

### 4.9. Machine Learning Analysis

In order to confirm the classical statistical results and establish a transition from correlation to prediction, machine learning (ML) analysis was performed with the help of JASP. In order to increase the reliability of the models and avoid overfitting, the whole study sample (*n* = 120) was split into two parts; 63% of the sample (*n* = 76) served as the Training Set to be used for training models, while 37% (*n* = 44) was retained as the Validation and Test Sets for checking the accuracy of the models.

Two different ML techniques were applied: The Random Forest ensemble technique was applied to build the hierarchical significance of studied biomarkers (*MEG3*, *NEAT1*, *miR-124*, *miR-146a*, IL-17, IL-6) in relation to the classification of the diseases. The biomarkers were ranked in accordance with their predictive value based on the “mean decrease in accuracy” criterion, which evaluates the impact of excluding a particular variable on the performance of the model. A Decision Tree model was applied to build a real-life clinical diagnosis model. This model reveals the most essential criteria of the threshold (root nodes and further branches) needed for stratification and separation of BD patients from healthy individuals.

## 5. Conclusions

In conclusion, the present study identified a characteristic molecular expression profile in patients with Behçet’s disease, characterized by the downregulation of the lncRNAs *MEG3* and *NEAT1* and the microRNAs *miR-124* and *miR-146a*, together with the upregulation of the pro-inflammatory cytokines IL-17 and IL-6. Collectively, these findings support a biologically plausible mechanistic framework in which reduced *NEAT1* expression may impair the regulatory activity of *miR-124* and *miR-146a*, thereby contributing to dysregulated STAT3- and TRAF6/NF-κB-associated inflammatory signaling. While the investigated biomarker panel demonstrated promising diagnostic discrimination between patients with Behçet’s disease and healthy controls, further validation in independent multicenter cohorts, including appropriate disease-control populations, together with functional mechanistic studies, will be required to establish their disease specificity and clinical utility.

## Figures and Tables

**Table 1:** Demographic characteristics of the study population.

Characteristics	BD Group (*n* = 60)	Healthy Control Group (*n* = 60)	*p*-Value
**Age (years)** **(Range)**	33.8 ± 7.9 (18–50)	35.17 ± 6.06 (21–43)	0.29
**Gender**			
**Male No. (%)**	52 (86.7%)	46 (76.7%)	0.157
**Female No. (%)**	8 (13.3%)	14 (23.3%)	

Data are expressed as mean ± standard deviation (range) for age and number (percentage) for gender. BD: Behçet’s disease. Differences between groups were analyzed using Student’s *t*-test for age and the chi-square test for gender distribution. *p* > 0.05 indicates no significant difference.

**Table 2:** Clinical manifestations and disease activity assessment of the BD cohort (*n* = 60).

**Patient’s Global Assessment** (Patient’s perception, 0:10)	6.48 ± 0.43	
**Physician’s Global Assessment** (Physician’s Perception, 0:10)	5.68 ± 0.423	
**Clinical Manifestations**		
	Present	Absent
**Headache No. (%)**	17 (28.3%)	43 (71.7%)
**Mouth Ulceration No. (%)**	25 (41.7%)	35 (58.3%)
**Genital Ulceration No. (%)**	8 (13.3%)	52 (86.7%)
**Erythema Nodosum No. (%)**	15 (25%)	45 (75%)
**Skin Pustules No. (%)**	8 (13.3%)	52 (86.7%)
**Joint Arthralgias No. (%)**	33 (55%)	27 (45%)
**Joint Arthritis No. (%)**	4 (6.7%)	56 (93.3%)
**Nausea/Vomiting/Abdominal Pain No. (%)**	13 (21.7%)	47 (78.3%)
**Diarrhea/BPR No. (%)**	5 (8.3%)	55 (91.7%)
**New Eye Involvement No. (%)**	35 (58.3%)	25 (41.7%)
**New Active Nervous System Involvement No. (%)**	5 (8.3%)	55 (91.7%)
**New Active Major Vessel Involvement No. (%)**	7 (11.7%)	53 (88.3%)
**BDCAF Patient’s Index Score (0:12)**	2.92 ± 0.237 (0–8)	
**BDCAF** (Transformed index score on interval scale, 0:20)	6.1 ± 0.364 (0–12)	
**BSAS/100**	27.48 ± 2.36 (0–80)	

Data are expressed as mean ± standard error of the mean (SEM) for numerical data or number (%) for categorical data. Ranges are presented in parentheses. BDCAF: Behçet’s Disease Current Activity Form; BPR: blood per rectum; BSAS: Behçet’s Syndrome Activity Score.

**Table 3:** Serum expression levels of non-coding RNA and cytokine concentrations in research cohort.

Variable	BD Patients (*n* = 60)	Control *(n* = 60)	Significance (*p* Value)
***MEG3***	0.41 ± 0.11	0.99 ± 0.004	<0.0001
***NEAT1***	0.12 ± 0.01	0.97 ± 0.017	<0.0001
***miR-124***	0.114 ± 0.016	0.98 ± 0.005	<0.0001
***miR-146a***	0.35 ± 0.038	0.985 ± 0.0062	<0.0001
**IL-17 (pg/mL)**	134.7 ± 7.92	39.62 ± 0.936	<0.0001
**IL-6 (pg/mL)**	67.81 ± 2.01	20.62 ± 0.455	<0.0001

Data are expressed as mean ± standard error of the mean (SEM). Statistical significance was assessed using Student’s *t*-test. A *p*-value < 0.05 was considered statistically significant. BD: Behçet’s disease; IL: Interleukin; *MEG3*: *Maternally Expressed Gene 3*; miR: microRNA; *NEAT1*: *Nuclear Paraspeckle Assembly Transcript 1*.

**Table 4:** Comparing serum biomarker levels and clinical characteristics in BD patients.

Clinical Manifestations		*MEG3*	*p*	*NEAT1*	*p*	*miR-124*	*p*	*miR-146a*	*p*	IL-17 (pg/mL)	*p*	IL-6 (pg/mL)	*p*
Gender	Female	0.093 (0.07–0.5)	0.5	0.1 (0.05–0.2)	0.6	0.05 (0.02–0.13)	0.3	0.14 (0.04–0.5)	0.5	109.7 (98.1–122.5)	0.4	52.9 (48.4–71.8)	0.09
	Male	0.09 (0.04–0.6)		0.09 (0.03–0.14)		0.08 (0.04–0.13)		0.3 (0.06–0.64)		119 (105.1–131.7)		69.6 (55.7–82.1)	
Headache	No	0.09 (0.04–0.4)	0.9	0.09 (0.03–0.17)	0.9	0.08 (0.04–0.14)	0.4	0.3 (0.06–0.57)	0.4	121.2 (105.3–131.4)	0.4	65.4 (52.9–82.1)	0.6
	Yes	0.09 (0.05–0.3)		0.08 (0.05–0.18)		0.07 (0.03–0.1)		0.17 (0.02–0.67)		111.9 (103.7–133.3)		75.4 (56.1–82.2)	
Mouth Ulceration	No	0.09 (0.04–0.5)	0.1	0.09 (0.03–0.17)	0.5	0.07 (0.04–0.15)	0.9	0.38 (0.05–0.65)	0.4	120.7 (106.8–131.9)	0.3	69.1 (55.7–82.1)	0.5
	Yes	0.07 (0.02–0.28)		0.09 (0.04–0.19)		0.08 (0.03–0.13)		0.3 (0.04–0.4)		115.8 (102.4–125.8)		65.4 (50–81.7)	
Genital Ulceration	No	0.09 (0.04–0.4)	0.7	0.08 (0.04–0.13)	0.3	0.07 (0.04–0.13)	0.8	0.3 (0.04–0.62)	0.8	119 (104.9–130.2)	0.7	65.9 (54.3–81.3)	0.3
	Yes	0.17 (0.04–0.56)		0.14 (0.05–0.23)		0.08 (0.03–0.14)		0.3 (0.07–0.6)		114.2 (106.1–204.8)		79.7 (54.2–87.1)	
Erythema Nodosum	No	0.1 (0.04–0.42)	0.1	0.09 (0.05–0.16)	0.7	0.07 (0.04–0.14)	0.8	0.3 (0.05–0.67)	0.5	115.8 (104.9–128.6)	0.5	63.4 (51.6–81.5)	0.1
	Yes	0.06 (0.02–0.3)		0.08 (0.03–0.26)		0.08 (0.06–0.13)		0.3 (0.04–0.5)		121.2 (105.3–145)		76.2 (59.3–82.1)	
Skin Pustules	No	0.09 (0.04–0.4)	0.5	0.09 (0.04–0.4)	0.5	0.08 (0.04–0.1)	0.2	0.3 (0.04–0.6)	0.1	118.1 (106–125.6)	0.7	64.4 (53.8–81.6)	0.3
	Yes	0.05 (0.02–0.8)		0.08 (0.03–0.1)		0.06 (0.02–0.1)		0.5 (0.2–0.09)		116 (85.175–150.7)		75.3 (61.2–82.1)	
Joint Arthralgias	No	0.09 (0.04–0.4)	0.4	0.09 (0.06–0.15)	0.8	0.1 (0.04–0.17)	0.08	0.3 (0.07–0.75)	0.2	111.8 (105–124.7)	0.07	65.4 (53–81.3)	0.7
	Yes	0.09 (0.03–0.3)		0.08 (0.04–0.17)		0.07 (0.03–0.11)		0.3 (0.04–0.5)		122 (106–146.2)		67.3 (54.8–82.7)	
Joint Arthritis	No	0.09 (0.04–0.4)	0.4	0.08 (0.04–0.17)	0.8	0.07 (0.04–0.13)	0.2	0.3 (0.04–0.6)	0.9	117 (105–126.4)	0.3	64.4 (53.8–81.3)	0.1
	Yes	0.25 (0.09–0.8)		0.1 (0.04–0.21)		0.11 (0.08–0.15)		0.27 (0.14–0.4)		205 (89.6–353.8)		82 (73.2–83.3)	
Nausea/Vomiting/Abdominal Pain	No	0.09 (0.04–0.43)	0.3	0.1 (0.05–0.14)	0.7	0.07 (0.04–0.1)	0.9	0.3 (0.06–0.5)	0.5	115.3 (105–125.8)	0.1	65.4 (54.3–81.8)	0.9
	Yes	0.09 (0.02–0.3)		0.07 (0.03–0.2)		0.08 (0.03–0.1)		0.1 (0.03–0.6)		124.7 (112–150)		75.4 (46–84.2)	
Diarrhea/BPR	No	0.09 (0.04–0.43)	0.4	0.09 (0.04–0.14)	1.0	0.08 (0.04–0.14)	0.7	0.3 (0.04–0.57)	0.5	119 (104–131.4)	0.8	66.4 (54.3–82.1)	0.9
	Yes	0.17 (0.1–0.27)		0.09 (0.03–0.24)		0.08 (0.05–0.24)		0.41 (0.09–0.68)		117.2 (104.1–250.3)		75.4 (52.6–80)	
New Eye Involvement	No	0.25 (0.06–0.6)	0.01 *	0.08 (0.03–0.12)	0.5	0.12 (0.03–0.16)	0.1	0.3 (0.07–0.68)	0.6	119 (103.1–128.8)	1.0	70.4 (58.2–82.1)	0.3
	Yes	0.07 (0.03–0.21)		0.09 (0.05–0.18)		0.06 (0.04–0.11)		0.3 (0.04–0.52)		116.8 (106–131.4)		59.3 (53.7–81.3)	
New Active Nervous System Involvement	No	0.09 (0.04–0.38)	0.3	0.08 (0.04–0.14)	0.2	0.07 (0.04–0.13)	0.2	0.3 (0.04- 0.57)	0.4	116.8 (104.9–126.6)	0.1	67.3 (53.7–82.1)	0.6
	Yes	0.25 (0.1–0.5)		0.12 (0.07–0.26)		0.15 (0.05–0.5)		0.26 (0.13–0.77)		124.7 (117.2–253.7)		63.4 (59–82.8)	
New Active Major Vessel Involvement	No	0.09 (0.04–0.4)	0.9	0.1 (0.05–0.17)	0.1	0.08 (0.04–0.13)	0.8	0.3 (0.05–0.64)	0.9	117.2 (104.8–125.4)	0.1	66.4 (54–81.5)	0.3
	Yes	0.09 (0.03–0.68)		0.03 (0.03–0.09)		0.08 (0.04–0.17)		0.24 (0.1–0.5)		126.6 (111.8–287.5)		70.4 (63.2- 84.2)	

Data are represented as median (25th–75th percentile). Statistical analysis was done using the Mann–Whitney U test. * *p* < 0.05 shows statistically significant differences. BPR: bleeding per rectum; IL: Interleukin; IQR: interquartile range; *MEG3*: *Maternally Expressed Gene 3*; miR: microRNA; NEAT1: *Nuclear Paraspeckle Assembly Transcript 1*.

**Table 5:** Correlations of non-coding RNAs, pro-inflammatory cytokines with BSAS and BDCAF scores of BD patients.

Variables	BDCAF (Transformed Index Score on Interval Scale, 0:20)		BDCAF (Patient’s Index Score, 0:12)		BSAS/100	
	r	*p*	r	*p*	r	*p*
***MEG3***	−0.125	0.34	−0.084	0.52	−0.052	0.69
***NEAT1***	0.115	0.383	0.07	0.59	0.015	0.9
***miR-124***	−0.231	0.076	−0.184	0.16	−0.176	0.179
***miR-146a***	−0.107	0.41	−0.124	0.34	−0.108	0.411
**IL-17 (pg/mL)**	0.238	0.067	0.274	0.03 *	0.125	0.34
**IL-6(pg/mL)**	0.092	0.48	0.13	0.321	0.11	0.36

Data are presented as Spearman’s rho correlation coefficient (*r*) and probability value (*p*). * *p* < 0.05 indicates a statistically significant correlation. BDCAF: Behçet’s Disease Current Activity Form; BSAS: Behçet’s Syndrome Activity Score; IL: Interleukin; *MEG3: Maternally Expressed Gene 3*; miR: microRNA; *NEAT1: Nuclear Paraspeckle Assembly Transcript 1*.

**Table 6:** Diagnostic performance of individual serum biomarkers and combined multi-marker panels in discriminating BD patients from healthy controls.

Biomarkers	AUC	*p*-Value	Cut-Off Value	Sensitivity	Specificity	PPV	NPV	Accuracy
***MEG3***	0.9	<0.0001	≤0.89	86.6%	90%	89.7	87.1	76.6%
***NEAT1***	0.83	<0.0001	≤0.75	83.33%	79.66%	80.6	80.6	63%
***miR-124***	0.85	<0.0001	≤0.755	83.3%	83.3%	83.3	83.3	66.6%
***miR-146a***	0.87	<0.0001	≤0.75	81.67%	91.67%	90.7	90.7	73.3%
**IL-17**	0.92	<0.0001	>59.2	93.3%	93.3%	93.3	93.3	86.6%
**IL-6**	0.91	<0.0001	>27.4	95%	95%	95	95	90%
***MEG3*** **with *NEAT1***	0.98	<0.0001	___	100%	98.3%	98.4	100	98.3%
***MEG3*** **with *miR-146a***	0.99	<0.0001	___	98.3%	96.6%	96.7	96.7	95%
***MEG3*** **with *miR-124***	1.000	<0.0001	___	100%	98.3%	92.3	92.3	98.3%
***miR-124*** **with *miR-146a***	1.000	<0.0001	___	100%	98.3%	92.3	92.3	98.3%

AUC: area under the receiver operating characteristic curve; PPV: Positive Predictive Value; NPV: Negative Predictive Value; IL: Interleukin; *MEG 3*: *Maternally Expressed 3*; *NEAT 1*: *Nuclear Paraspeckle Assembly Transcript 1*; miR: microRNA. *p*-values are considered highly significant at <0.0001. Note: The cut-off values for combined panels (indicated by ___) are based on predicted probability scores derived from binary logistic regression and do not correspond to a single concentration unit.

**Table 7:** Positivity rates of the biomarkers studied across the investigated groups.

Biomarker	BD Patients (*n* = 60)	Controls (*n* = 60)	χ^2^	*p*-Value
**IL-17**			90.13	<0.0001
**Positive (>59.2)**	56 (93.3%)	4 (6.7%)		
**Negative (≤59.2)**	4 (6.7%)	56 (93.3%)		
**IL-6**			97.3	<0.0001
**Positive (>27.4)**	57 (95%)	3 (5%)		
**Negative (≤27.4)**	3 (5%)	57 (95%)		
***MEG3***			70.45	<0.0001
**Positive (≤0.89)**	52 (86.7%)	6 (10%)		
**Negative (>0.89)**	8 (13.3%)	54 (90%)		
***NEAT1***			48.2	<0.0001
**Positive (≤0.75)**	50 (83.3%)	12 (20%)		
**Negative (>0.75)**	10 (16.7%)	48 (80%)		
***miR-124***			53.3	<0.0001
**Positive (≤0.75)**	50 (83.3%)	10 (16.7%)		
**Negative (>0.75)**	10 (16.7%)	50 (83.3%)		
***miR-146a***			64.86	<0.0001
**Positive (≤0.75)**	49 (81.7%)	5 (8.3%)		
**Negative (>0.75)**	11 (18.3%)	55 (91.7%)		

χ^2^: chi-square test value; BD: Behcet’s disease; IL: Interleukin; *MEG3: Maternally Expressed Gene 3*; miR: microRNA; *NEAT1*: *Nuclear Paraspeckle Assembly Transcript 1*. Positive cases for IL-17/IL-6 are those with levels *above* the cut-off. Positive cases for RNAs (*MEG3*, *NEAT1*, miRNAs) are those with expression levels *below* the cut-off (indicating downregulation).

**Table 8:** Multivariate binary logistic regression analysis identifying independent predictors of BD.

Predictor	B	S.E.	Wald	*p*-Value	Exp(B) (OR)
**Model 1: *MEG3***					
**Age**	−0.001	0.031	0	0.986	0.999
**Gender**	0.822	0.528	2.421	0.12	2.274
***MEG3***	−2.418	0.533	20.546	<0.001	0.089
**Model 2: *NEAT1***					
**Age**	−0.12	0.148	0.658	0.417	0.887
**Gender**	6.854	5.496	1.555	0.212	947.807
***NEAT1***	−16.039	7.6	4.454	0.035	0.035
**Model 3: *miR-124***					
**Age**	0.146	0.409	0.127	0.722	1.157
**Gender**	3.896	10.72	0.132	0.716	49.189
***miR-124***	−19.634	9.923	3.915	0.048	0
**Model 4: *miR-146a***					
**Age**	−0.197	0.102	3.76	0.053	0.821
**Gender**	3.111	2.309	1.815	0.178	22.438
***miR-146a***	−23.987	6.214	14.899	<0.001	0

B: regression coefficient; S.E.: standard error; Wald: Wald chi-square statistic; Exp(B): Odds Ratio (OR).

**Table 9:** The sequences of primers used in qPCR assays for the chosen lncRNAs.

***NEAT1***	Forward 5′-TGGCTAGCTCAGGGCTTCAG-3′
	Reverse 5′-TCTCCTTGACCAAGGAGCGG-3′
***MEG3***	Forward 5′-CTGCCCATCTACACCTCACG-3′
	Reverse 5′-CTCTCCGCCGTCTGCGCTAG-3′
***GAPDH***	Forward 5′-CCCTTCATTGACCTCAACTA-3′
	Reverse 5′-TGGAAGATGGTGATGGGATT-3′

*GAPDH*: glyceraldehyde-3-phosphate dehydrogenase (internal control); *MEG3*: Maternally *Expressed 3; NEAT1*: *Nuclear Paraspeckle Assembly Transcript 1*.

## Data Availability

The datasets generated and/or analyzed during the current study are not publicly available due to privacy and ethical restrictions associated with human participant data but are available from the corresponding author upon reasonable request.
